# Effect of long anther dehiscence on seed set at high temperatures during flowering in rice (*Oryza sativa* L.)

**DOI:** 10.1038/s41598-019-56792-2

**Published:** 2019-12-30

**Authors:** Tsutomu Matsui, Toshihiro Hasegawa

**Affiliations:** 1 Faculty of Applied Biological Sciences, Gifu University, Gifu, 501-1193 Japan; 2 NARO Institute for Agro-Environmental Sciences, 3-1-3 Kannondai, Tsukuba, Ibaraki 305-8604 Japan; 3 Present Address: NARO Tohoku Agricultural Research Center, 4 Shimokuriyagawa Azaakahira, Morioka, Iwate 020-0198 Japan

**Keywords:** Plant sciences, Plant ecology, Plant stress responses

## Abstract

Countermeasures that can mitigate the global warming impact on rice production are needed. The large dehiscence of anther for pollen dispersal is one trait that shows tolerance of seed set to high temperatures under the global warming. The aim of this study is to determine the effect of long anther dehiscence on high temperature tolerance. Seven chromosome segment substitution lines and the seed parent with the different dehiscence lengths were subjected to high daytime temperatures. Elongation of dehiscence formed at the base of anther (BDL) by 100 µm mitigated the occurrence of high temperature induced sterility by 20% and improved tolerance to the high temperature by 0.66 °C. Relationship between the seed set and BDL was well explained by pollination, showing that quantitative information provided in the present experiment is reliable. The information is expected to be used in estimation of global warming impact and making countermeasures for it.

## Introduction

We must evaluate the large anticipated impact of global warming on agricultural production and carefully consider countermeasures that may contribute to the survival of humans^1^. In rice production, temperatures above 35 °C at flowering induce floret sterility^2–4^. Crop simulation models show that warming may induce floret sterility and reduce yields^5^, even in temperate regions such as Japan^6^. One effective countermeasure to mitigate the adverse effects of global warming is to adopt high temperature-tolerant cultivars^7^. However, the extent to which we can improve the high-temperature tolerance of rice is unclear.

Although the genetic and physiological mechanisms of high temperature-induced floret sterility are unclear, the tolerance to high temperatures varies by more than 3 °C in rice^2^. One cause of the variation in tolerance to high temperatures is the difference in the size of anther dehiscence at flowering^8^. A rice floret once open in the morning for around one hour normally. The rice floret is most sensitive to high temperatures at the flowering time and the high temperatures disturb the pollination^2^. Poor pollination is the main direct cause of high temperature induced floret sterility^2,3^. A long dehiscence at the base of the anther helps the drop of pollen grains onto the stigmas and thereby ensures stable pollination under high temperatures^8^, as well as under the normal condition^9^. Therefore, the long basal dehiscence of anthers is a candidate character for breeding objective in rice.

The effect of long basal dehiscence on pollination and seed set has been examined with various cultivars under controlled^8,9^ and field^11,12^ conditions. The correlations between the anther dehiscence, pollination stability and seed set across the wide genetic variations observed in the former studies show strong relationships between them. However, the quantitative relationships among these characters may be difficult to disentangle because of the noise generated by their high genetic diversity.

Our purpose in this study was to confirm the role of long anther dehiscence in high temperature tolerance and quantify the effect of long anther dehiscence on tolerance to high temperature at flowering in rice with simple genetic and environmental background. We used chromosome segment substituted lines (CSSLs) with various levels of dehiscence at both the anther apex and base to create a simple genetic background. The pot grown rice were subjected to three high day temperatures (35, 37, 39 °C) at flowering for three days. Linear regression analyses were conducted to quantify the relationships between anther dehiscence, pollination, and seed set.

## Results

### Anther dehiscence and indehiscence are correlated with pollination and seed set

The anther dehiscence traits, ADL (apical dehiscence length, length of anther dehiscence formed at apical part of theca for pollen dispersal), BDL (basal dehiscence length, that at basal part of theca), IA (percentage of indehiscence at the apical part of theca), and IB (that at the basal part of theca) were significantly correlated with pollination and seed set (Table 1). At 35 °C day temperature, BDL was closely correlated with SS (percentage of seed set of panicle that flowered under high temperatures), GPM5 (percentage of florets having 5 or more germinated pollen grains on the stigma after anthesis), and TPM10 (percentage of florets having 10 or more total (germinated and ungerminated) pollen grains on the stigma). ADL was also significantly correlated SS and GPM5. At 37 °C, BDL was significantly correlated with SS, while ADL was significantly correlated with GPM5 and TPM10. At 39 °C, ADL and BDL were significantly correlated with SS but not with GPM5 and TPM10. IA and IB were significantly correlated with GPM5 and TPM10 at 37 °C and 39 °C but not at 35 °C. IB was also significantly correlated with SS at 39 °C. At 37 °C where the range of SS was largest (data not shown), the SS was approximated by the regression *Y* = 0.00952*X* − 4.08 (1), where *Y* is logit transformed SS and *X* is BDL (µm). Confidence intervals of partial regression coefficients of *X* at 95% level was 0.00431–0.01472.

**Table 1:** Correlation coefficients (r) between morphological traits of dehisced anthers, and seed set and pollination at three daytime temperatures.

Temperature (°C)	Anther trait	SS	GPM5	TPM10
35	ADL	0.751*	0.714*	0.693 ns
	BDL	0.936***	0.950***	0.944***
	IA	−0.601 ns	−0.480 ns	−0.517 ns
	IB	−0.648 ns	−0.595 ns	−0.633 ns
37	ADL	0.455 ns	0.878**	0.855**
	BDL	0.893**	0.640 ns	0.696 ns
	IA	−0.365 ns	−0.792*	−0.778*
	IB	−0.439 ns	−0.788*	−0.755*
39	ADL	0.759*	0.512 ns	0.568 ns
	BDL	0.721*	0.661 ns	0.672 ns
	IA	−0.531 ns	−0.854**	−0.870**
	IB	−0.761*	−0.817*	−0.849**

ADL, length of dehiscence formed at the apical part of the theca; BDL, length of dehiscence formed at the basal part of the theca; IA, percentage of indehiscence of the apical part of the theca; IB, percentage of indehiscence of the basal part of the theca; GPM5; percentage of florets having 5 or more germinated pollen grains on the stigma after anthesis; TPM10, percentage of florets having 10 or more pollen grains (including germinated and ungerminated pollen grains) on the stigma after anthesis; SS, percent seed set at maturity. Data for % florets and SS were arcsine- and logit-transformed, respectively, before the correlation analysis with length of dehiscence. ^*^P < 0.05; ^**^P < 0.01; ^***^P < 0.001; ns, not significant (n = 8).

### Calculations for T_75_ and T_50_ and correlation with anther dehiscence

For the different genotypes, the logit-transformed SS values across temperatures were well approximated with a linear regression model estimated by analysis of covariance (Table 2).

**Table 2:** Logit-transformed seed set values, the intercepts of linear regression models for the relationship between the day temperature and logit transformed seed set, and T_75_ and T_50_ values (temperature at which seed set is reduced to 75% and 50%, respectively) for the eight genotypes.

Genotype	Logit-transformed seed set rate under different daytime temperatures (°C)			Intercept (C)	T_75_	T_50_
	35	37	39			
NKC07	1.99	−0.03	−3.03	47.70	35.9	36.7
NKC13	2.97	0.01	−1.15	48.66	36.6	37.5
NKC16	1.18	−1.54	−4.74	46.35	34.8	35.7
NKC21	1.66	−0.43	−4.07	47.11	35.4	36.3
NKC22	0.70	−0.60	−3.42	46.95	35.3	36.1
NKC25	1.45	−0.73	−3.71	47.06	35.4	36.2
NKC45	2.85	0.69	−3.05	48.22	36.3	37.1
Nipponbare	1.72	−0.23	−3.87	47.27	35.5	36.4

Logit transformed seed sets were explained by daytime temperatures using a regression model with the same slope estimated by analysis of covariance, *Y* = –1.299*X* + C, where *Y* is the logit transformed seed set, *X* is the temperature and C is constants for genotypes. (*R*^*2*^ = 0.964, *P* < 0.00001, n = 24). T_75_ and T_50_ were estimated with this equation.

With this linear regression model, T_75_ (the day temperature at which SS decreased to 75%) and T_50_ (the day temperature at which SS decreased to 50%) for each genotype were calculated. The T_75_ and T_50_ ranged from 34.8 to 36.6 °C and from 35.7 to 37.5 °C, respectively. The ADL, BDL and IB significantly correlated with T_75_ andT_50_ across the genotypes (Table 3).

**Table 3:** Summary of single regression analysis of anther dehiscence and estimated day temperature at which seed set decrease to 75% or 50%.

Variable of anther morphology	*R*^2^	Regression coefficient	95% confidence interval for regression coefficient	
			Lower limit value	Upper limit value
ADL	0.570*	0.00501*	0.00935	0.00066
BDL	0.864***	0.00801***	0.01119	0.00483
IA	0.323 ns	—	—	—
IB	0.512*	−1.878*	–0.047	–3.709

ADL, average length of dehiscence formed at the apical part of the anther for three daytime temperatures; BDL, average length of dehiscence formed at the basal part of the anther for three daytime temperatures; IA, average percentage of indehisced theca of the apical part for three day temperature; IB, average percentage of indehisced theca of the basal part for three day temperatures. ^*^P < 0.05; ^***^P < 0.001; ns, not significant (n = 8).

The T_75_ and T_50_ values of the genotypes were well explained with the following multiple regression equations with BDL and IB (Table 4):

2 $$Y=0.00657{X}_{1}-0.896{X}_{2}+C,\,C=33.58\,{\rm{f}}{\rm{o}}{\rm{r}}\,{{\rm{T}}}_{75},\,34.43\,{\rm{f}}{\rm{o}}{\rm{r}}\,{{\rm{T}}}_{50}$$

where *X*_1_, and *X*_2_ are BDL (µm), and logit transformed IB, respectively. Confidence intervals of partial regression coefficients of *X*_1_ at 95% level were 0.00407–0.00906. The ADL was not adopted because of low partial *F*-value in process of forward selection of the multiple regression analysis.

**Table 4:** Summary of multiple regression model for temperatures at which seed set percentage decrease to 75% (T_75_) and to 50% (T_50_) with anther dehiscence characteristics as explanatory variables, and with different intercepts for the temperatures.

Variable	Partial regression coefficient for variables and intercept	Standardized partial regression coefficient	Partial correlation coefficient
**Explanatory variables**			
BDL_av_	0.00657**	0.7617	0.95
IB_av_	−0.8958*	−0.3413	−0.805
**Intercept**			
for T_75_	33.58***		
for T_50_	34.43***		

For the equation of multiple regression, *R*^2^ = 0.8637, and *P* = 0.0005 (n = 16). BDL, average length of dehiscence formed at basal part of anther for three day temperatures (µm); IB, average percentage of indehiscence at the basal part of the anther for three day temperatures. ^*^P < 0.05, ^**^P < 0.01^, ***^P < 0.001. ADL was not adopted in process of forward selection with F-in value of 9.0. IA was not used as a variable in this analysis because of high correlation with IB of which correlation coefficient with T_75_ and T_50_ were higher than IA.

### Regressions between pollination and seed set

**Figure 1:**
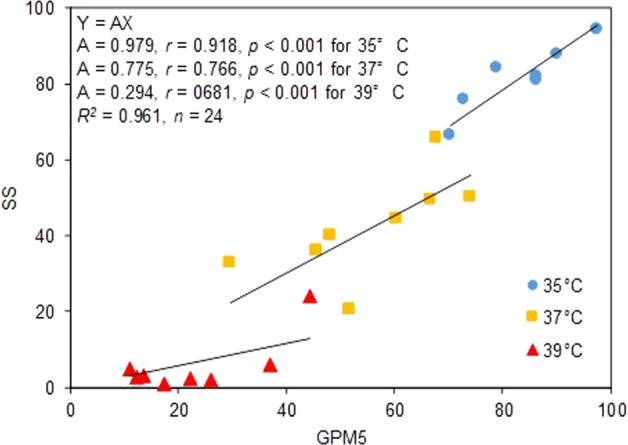
The relationship between the percentage of florets with five or more germinated pollen grains (GPM5) and the % seed set (SS). SS was well approximated by GPM5 using a general linear model with separate slope design, Y = AX, where X is the GPM5 and Y is the SS. *r*, partial correlation coefficient; *R*^2^, Coefficient of determination for the model.

**Figure 2:**
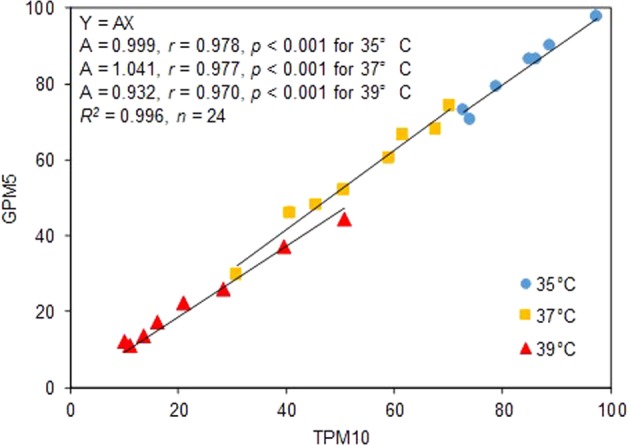
The relationship between the percentage of florets with 10 or more total pollen grains on the stigma (TPM10) and the percentage of florets with five or more germinated pollen grains (GPM5). GPM5 was well approximated by TPM10 using a general linear model with separate slope design, Y = AX, where X is the TPM10 and Y is the GPM5. *r*, partial correlation coefficient; *R*^2^, Coefficient of determination for the model.

The regression model for the relationship between SS and GPM5 passing origin and with each slope for each day temperature was significant across the genotypes (Fig. 1). Thus, the SS of the genotypes was well explained by GPM5. Similarly, the regression models for the relationship between GPM5 and TPM10 passing origin and with each slope for each day temperature was significant across the genotypes (Fig. 2). The GPM5 of the genotypes was, in turn, well explained by TPM10. The slopes ranged from 0.923 to 1.041.

## Discussion

From the regression equations, we can quantify the effect of elongation of the anther dehiscence on the seed set. Based on the Eq. (1), *Y* = 0.00952*X* − 4.08 for the regression line between the BDL (*X*) and logit-transformed % of seed set at 37 °C (*Y*), the elongation of BDL by 100 μm would improve the seed set by 0.952 in logit-transformed value that is approximately equivalent to 20% around 50% of seed set. Confidence intervals of partial regression coefficients of *X* at 95% level, 0.00431–0.01472 is approximately equivalent to 11–35% improvement by 100 μm elongation around 50% of seed set. Tian *et al*.^11^ report the regression equation *Y* = 0.155*X* + 15.79, where *Y* is the seed set (%) and *X* is the BDL (µm), based on data from ten hybrid rice cultivars in China; and Zhao *et al*.^12^ report the equation *Y* = 0.0674*X* + 56.21, where *Y* is the seed set (%) and *X* is BDL (µm), based on data from six genetically diverse cultivars. These reports suggest approximate increases of 15.5 and 6.7% in seed set with an increase in BDL of 100 µm. Considering variations in tolerance and degree of occurrence associated with genetic and environmental factors, present results agree well with these past reports.

The T_75_ and T_50_ values of the genotypes were well explained by multiple regressions with BDL and IB (Table 2). Based on the partial regression coefficients in the Eq. (2), the elongation of basal dehiscence by 100 µm would improve the T_75_ and T_50_ by 0.66 °C (95% confidence interval, 0.41–0.91 °C). Tazib *et al*.^13^ detected four major QTLs for BDL and confirmed the effect of one with CSSLs. Our results support the validity of breeding with the QTLs. We can translate the additive effect of QTL on anther length into high temperature tolerance with 0.66 °C per 100 µm and estimate the breeding effect using the alleles. We also recommend that 0.66 °C per 100 µm for BDL should be a guide for the estimation of high temperature-tolerant resources in a survey and for the adoption of genotypes with long BDL. Present multiple regression analysis revealed that indehiscence of anther was also important factor of high temperature tolerance. Matsui *et al*.^14^ and Matsui & Omasa^15^ showed that the difference in development of lacuna formed between septum and stomium participate the tolerance of dehiscence of anther under high temperature. Easily dehiscent characteristics of anther with the well-developed lacuna seems important for the high temperature tolerance as well as long BDL.

ADL was not adopted as an explanatory variable in forward selection with F_IN_ value of 9.0, although it significantly correlated with T_50_ and T_75_ with single regression analysis. From the regression coefficient, the elongation of ADL by 100 µm improve the tolerance by 0.5 °C (95% confidence interval 0.006–0.90 °C) (Table 3). However, the rejection by the multiple regression suggest that the contribution of ADL to tolerance is smaller than that of BDL.

The SS was explained by GPM5 at each temperature across genotypes with straight lines all passing through the origin with slopes of 0.979 for 35 °C, 0.775 for 37 °C, and 0.294 for 39 °C (Fig. 1). Because rice seed set generally fails when the number of germinated pollen grains on the stigma is less than 5 or 10^2,3^, we assume that SS is proportional to GPM5 and adopted a linear model passing through the origin. The strong proportional relationship between SS and GPM5 at 35 and 37 °C supports the theory of Satake and Yoshida^2^: those authors proposed that the primary cause of high temperature induced-floret sterility below 37 °C, particularly at 35 °C, is the decrease in number of germinated pollen grains, less than five or ten, on the stigma. At 39 °C, the slope of the regression line is approximately 0.3. This low slope indicates that the processes after germination such as pollen tube elongation etc. are disturbed by the high temperature and thus five germinated pollen grains are not enough for seed set under 39 °C. Moreover, we found that the partial correlation coefficient (*r*) is not sufficient to explain the difference in tolerance between genotypes at this temperature. The low slope and *r* value at the highest temperature is consistent with the report of Matsui *et al*.^3^, who showed the importance of the process after pollen germination in the tolerance to the extreme high temperatures. We found the GPM5 almost coincided TPM10 in all temperatures (Fig. 2), indicating that instability in the germination was primarily caused by instability in pollination. This also coincide the theory of Satake and Yshida^2^ that poor pollination is the main cause of poor germination caused by high temperature at flowering.

The TPM10 and GPM5 were significantly correlated with BDL and ADL across genotypes at 35 and 37 day temperatures, respectively, and with IA and IB at the two higher temperatures (Table 1). Our results are consistent with previous reports that showed correlations between BDL and pollination under field conditions^11,12^, under controlled conditions^8^, between BDL and ADL and pollination under field conditions^16^, and between BDL and anther indehiscence and pollination under controlled environments^10^. We found TPM10 was correlated with IB and IA across genotypes at 37 and 39 °C, whereas TPM10 was correlated with BDL at 35 °C and with ADL at 37 °C. The dominant factor affecting pollination seems to shift from the anther dehiscence lengths to indehiscence with increase in day temperature.

Although the correlations between ADL and TPM10 or GPM5 and between BDL and TPM10 or GPM5 were not significant at 39 °C, both ADL and BDL were significantly correlated with SS at 39 °C. Song *et al*.^17^ report that half-value period of pollen germination ability after anther dehiscence was 6 minutes for a rice cultivar (Minghui 63). We propose that a long BDL and ADL may contribute to pollen viability affecting the process after pollen germination and thus seed set through smooth pollen dispersal and supplying fresh pollen onto stigma at the start of anther dehiscence. This may be a reason why ADL and BDL significantly correlated with seed set under extreme high temperatures (39 °C) where correlations between ADL or BDL and the pollination stability parameters were not significant.

## Conclusion

In this study we quantified the effect of elongation of anther dehiscence on high temperature induced floret sterility with confirming its mechanism. Elongation of the dehiscence formed at basal part of anther by 100 µm would improve the tolerance of floret to day high temperature by 0.66 °C through increasing the stability of pollination. Long basal anther dehiscence is important characteristics to improve the high temperature tolerance in rice. The quantified effect of long anther dehiscence is expected to be used in estimation of counter measures against global warming.

## Methods

### Plant materials

**Figure 3:**
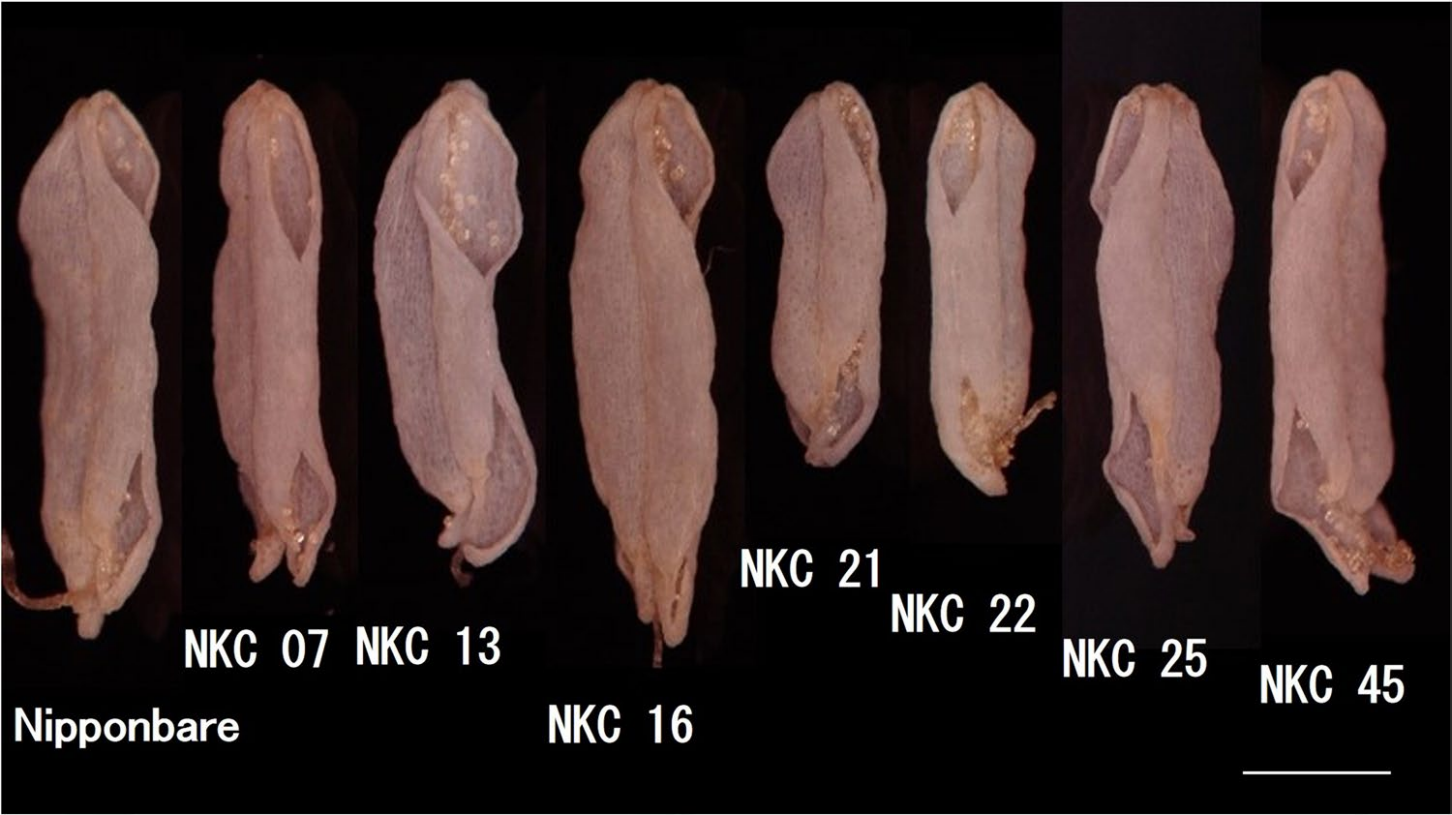
Variation in the morphology of dehisced anthers among CSSLs and the seed parent (Nipponbare) used in this experiment. Scale bar represents 500 µm.

To minimize the effects of factors other than anther dehiscence traits and to achieve the accurate estimation of effect of anther dehiscence length on high temperature tolerance, CSSLs that have the same genetic background were used. Seven lines selected from series of Nipponbare (variety with long anther dehiscence)/Kasalath (variety with short anther dehiscence) CSSLs and Nipponbare (the seed parent of the CSSLs) were used (Fig. 3).

The series of CSSLs are consisted of 54 CSSLs derived from crossing Nipponbare × Kasalath followed by back crossing with Nipponbare (Nipponbare genetic background). The seven CSSLs (NKC07, NKC13, NKC16, NKC21, NKC22, NKC25 and NKC45) were selected to cover wide variation in ADL and BDL depending on the preliminary morphological research of dehisced anther of the CSSLs. The genotypes were obtained from the National Institute of Agrobiological Sciences Resources (http://www.nias.affrc.go.jp/index_e.html) and the genotype data are open (https://www.rgrc.dna.affrc.go.jp/ineNKCSSL54.html).

Rice seeds were sown in 2011 on 25 April for NKC07, NKC25 and NKC45, on 17 May for NKC16, NKC21, NKC22 and Nipponbare, and on 3 June for NKC13. Seedlings, each at the 5th leaf stage, were transplanted in a circular pattern into 4-L pots, with 20 seedlings per pot^18^. The pots contained 3.0 kg equivalent of air-dried soil. The soil was a sandy loam with pH 7.64. In each pot, 0.5 g of N (58% from CO(NH_2_)_2_, 37% from (NH_4_)_2_HPO_4_, and 5% from (NH_4_)_2_SO_4_), 0.5 g of P_2_O_5_, and 0.5 g of K_2_O of slow-release fertilizer (Emu-coat 777, National Federation of Agricultural Cooperative Associations, Tokyo) were provided as a basal dressing before transplanting. After transplanting, the seedlings grew outside in the pots under submerged soil conditions. To obtain uniform panicles on the main culms, the tillers were removed as they appeared. For each genotype, nine pots with uniformly sized seedlings were randomly divided into three treatments with three pots each. The heading of rice occurred from 6 to 29 August.

### Treatments

The high temperature treatments were established in three growth chambers (LPH-500-LC, NIPPON MEDICAL & CHEMICAL INSTITUTE, Osaka, Japan). Pot-grown rice were moved into each chamber after we observed flowering and exposed to 35, 37, or 39 °C for seven hours (0900 to 1600 h) for three consecutive days. Genotypes were treated one by one in order to their heading dates. The nighttime temperature (1800 to 0700 h) was 25 °C for all treatments. The relative humidity was 70% during the day and 90% at night. From 0700 to 0900 h and from 1600 to 1800 h, air temperature and humidity were changed stepwise, either up or down, respectively. Sensors (HMP45A, Vaisala, Helsinki, Finland) set in ventilated double cylinders (length: 45 cm, inner diameters: 7 and 4 cm, wind speed: 2.15 m s^−1^) monitored the temperatures and humidity in growth chambers at height of panicle center, and daytime temperatures were carefully adjusted to the set values of 35, 37, and 39 °C based on the monitored temperatures. In each growthchamber, three pots were placed on the center of chamber in line perpendicularly to the wind direction. Air temperature and humidity were measured on the windward side of center pot. The distance between the opening of cylinder and the nearest panicle was set to be 10 cm. Mean day and night temperatures in each day were within +0.23 to −0.37 °C and +0.10 to −0.52 °C of set temperatures, respectively, during treatment period (August 6 to August 31). Mean relative humidity in the daytime and the nighttime in each day were within +4.9 to −2.4% and +1.2 to −5.1% of set value, respectively. Temperature and relative humidity recorded every 2 minutes in daytime ranged +0.86 to −0.68 °C and +6.5 to −11.6% around the mean value, respectively, during the treatment period. Air temperatures were fluctuated around the mean values and standard deviation of recorded day temperatures were 0.29 °C for 39 °C day temperature treatment, 0.21 °C for 37 °C treatment, and 0.23 °C for 35 °C treatment. The photon flux density and the photosynthetic photon flux density in the chambers (measured at the bottom of the chambers without plant materials) during the day (0600 to 1800 h) were approximately 203 and 186 μmol m^−2^ s^−1^, respectively. The pots with rice plants were carried into and out of the chambers at approximately 1800 h.

### Measurements

Each panicle was tagged with its heading date. The panicles on which one or two florets flowered on the day before the start of high temperature treatments were used for the seed set test. Seed set was examined by manual inspection of ovarian development with a forefinger at maturity. The percentage of seed set was calculated for each panicle (number of florets with swelled ovary/number of total florets × 100), and the average of the panicles in a pot was used as the replicate percentage of seed set (SS). At least two panicles from each pot were examined for the test.

**Figure 4:**
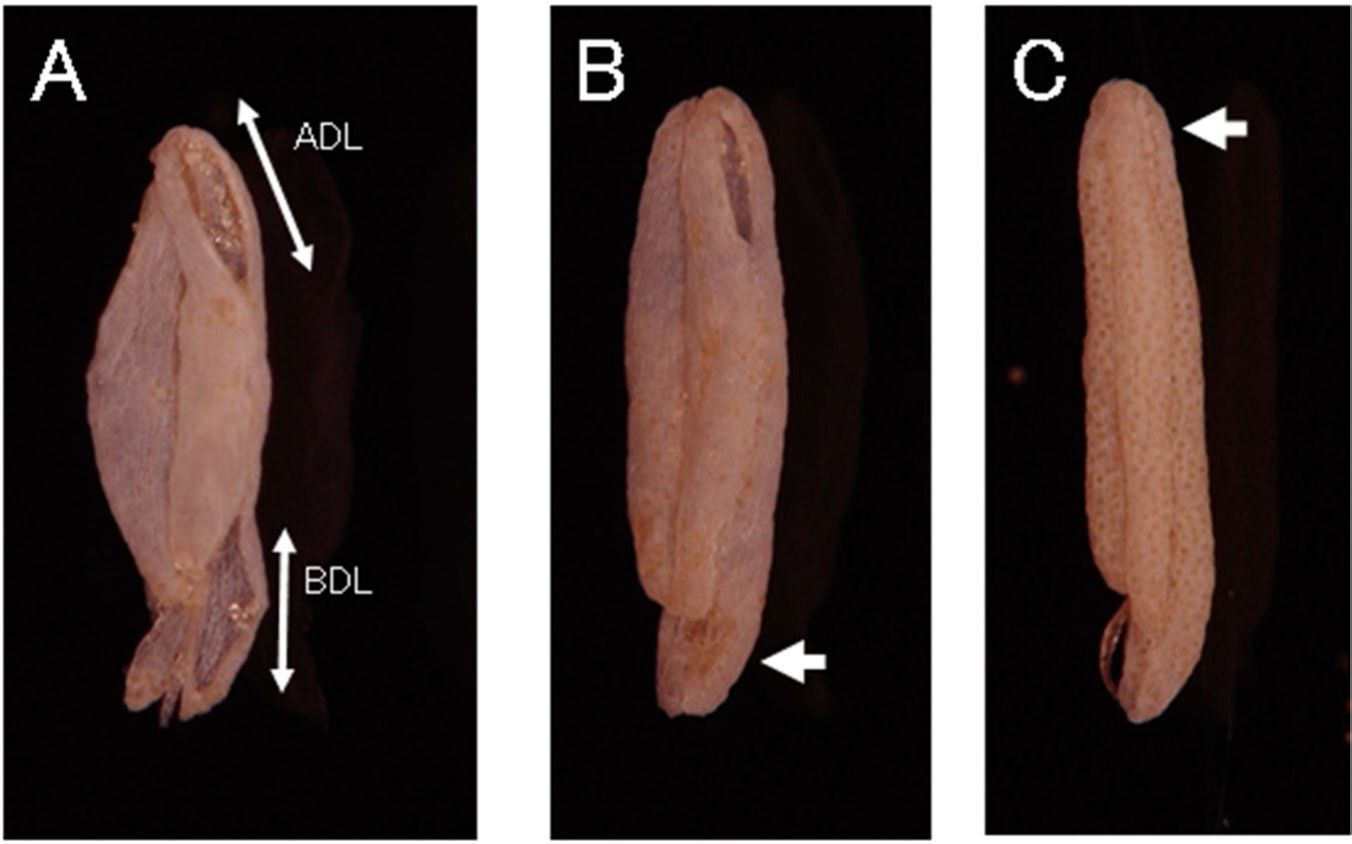
(**A**) Dehiscence for pollen dispersal formed on the apical and basal parts of theca (NKC 07). ADL, length of dehisce formed at apical part; BDL, length of dehiscence formed at basal part of theca. (**B**) Partially dehisced anther (NKC 07). Arrow indicates indehiscence occurred at basal part of theca. (**C**) Indehisced anther. Arrow indicates indehiscence occurred at apical part of theca.

During the treatments, florets that flowered on a particular day were sampled at 1600 from the panicles other than those for seed set test. The anthers were immediately detached and naturally air dried in Petri dishes. The dried anthers were used for measurements of length of dehiscence formed at the basal (BDL) and apical (ADL) parts on the anthers using digital microscope (KH-7700, Hirox, Tokyo, Japan). Before measuring the lengths of dehiscence, the percentage of indehiscence at the apical (IA) and basal (IB) parts of a theca was first examined (Fig. 4). Four randomly selected anthers from a floret were measured.

Following the detachment of anthers, the stigmas were sampled. The stigmas were stained with cotton blue solution, and the number of germinated and total (germinated + ungerminated) pollen grains on the stigmas was counted with an optical microscope at 100× magnification. For successful seed set, rice generally requires more than five or ten germinated pollen grains or more than 10 or 20 total pollen grains (germinated + ungerminated pollen grains) on the stigma^1,2^. Therefore, as indices of pollination stability, the percentage of florets having ten or more total pollen grains (TPM10) or having five or more germinated pollen grains (GPM5) on the stigma after anthesis was calculated. For the observations of anther dehiscence and pollen grains on stigmas, three florets per pot (nine total florets for each treatment) were sampled on each of the three days of high temperature treatment. The average dehiscence of the three florets per pot represented the score of each replicate.

### Data analyses

Analysis of variance (ANOVA) was conducted on anther dehiscence, pollination, and seed set with the temperature as a block, genotype as a main factor and number of days during which florets were exposed to treatments (length of treatment) as a sub-plot factor. For the traits that were found significant, we conducted the Tukey’s HSD test and regression analysis to quantify the effect of anther dehiscence on seed setting under different temperature regimes and to confirm the path from the anther dehiscence to heat tolerance. Since the effect of length of treatment was not significant for the morphological variables (i.e., ADL, BDL, IA and IB) and pollination variables (i.e., GPM5 and TPM10) in the ANOVA, the average of the mean values for the three days was used for them in the regression analysis. Moreover, for the morphological variables in which the interactions between temperature, length, and genotype were not significant in the ANOVA, the average of the mean values at the three daytime temperatures was used for the mean of each genotype in the regression analysis.

The T_75_ and T_50_ were calculated for each genotype depending on the linear regression model between the logit-transformed seed set rate and daytime temperature estimated by the analysis of covariance. The relationships between T_75_ or T_50_, the lengths of dehiscence formed at apical and basal parts on anthers, and the percentage of indehiscence at the basal and apical parts of thecae were analyzed with multiple regressions. We adopted forward selection of variables with F_IN_ and F_OUT_ (partial *F* value for entry and stay of variable) of 9.0 and 8.5, respectively.

The data for SS rate were analyzed after logit transformation. Because the data for TPM10, GPM5, IA, and IB included values of 0, the data were arcsine-transformed before analysis. The relationships between SS, GPM5, and TPM10 were, however, analyzed using a general linear model with separate slope design without this transformation. The statistical analyses were conducted using STATISTICA ver. 10 (StatSoft, Inc., Tulsa, OK, USA).
